# Right Ventricular Fibrosis With Pulmonary Arterial Hypertension

**DOI:** 10.31083/RCM42395

**Published:** 2025-12-19

**Authors:** Xinrui Li, Peng Liu, Yongnan Li, Yang Liu, Wei Hao, Ping Jin, Rongzhi Zhang

**Affiliations:** ^1^Department of Anesthesiology, The Second Hospital & Clinical Medical School, Lanzhou University, 730000 Lanzhou, Gansu, China; ^2^Department of Cardiac Surgery, The Second Hospital & Clinical Medical School, Lanzhou University, 730000 Lanzhou, Gansu, China

**Keywords:** pulmonary hypertension, right ventricular, fibrosis, clinical relevance, right heart failure

## Abstract

Pulmonary hypertension (PH) is a progressive disease caused by structural and functional changes in the pulmonary vasculature resulting from diverse etiologies. PH ultimately leads to increased right ventricular (RV) afterload, RV hypertrophy, fibrosis, and right heart failure (RHF). Moreover, RV fibrosis initially serves as a protective mechanism against pressure overload-induced RV dilatation, but eventually progresses to excessive fibrosis, which impairs cardiac function. This review explores the relationship between RV fibrosis and RV function in PH patients, examines the clinical relevance of this relationship, evaluates techniques for quantifying RV fibrosis, and presents potential therapeutic strategies aimed at preserving right heart function in PH patients.

## 1. Introduction

Pulmonary hypertension (PH) is defined as a mean pulmonary artery pressure (mPAP) of ≥20 mmHg at rest, as measured by right heart catheterization (RHC). The World Health Organization (WHO) classifies PH into five groups based on etiology: pulmonary arterial hypertension (PAH), PH secondary to left heart disease, lung disease, pulmonary artery obstructions, and multifactorial mechanisms [1, 2]. Among these, PAH is one of the most severe subtypes of PH.

The prevalence of PH is estimated to be approximately 1% worldwide, with a higher prevalence in individuals aged >65 years and those with cardiopulmonary complications [2]. PH is also more common in women, with a female to male prevalence of 4.3:1 [3]. The survival of adult patients with PH depends on age, severity of disease, underlying etiology, and the availability of evidence-based treatments [4]. PH is not easily curable and is therefore associated with a high mortality rate.

Right ventricular (RV) function is an important determinant of survival. Initially, the RV undergoes adaptive changes due to pressure overload. As the pulmonary artery pressure increases over time, maladaptive RV hypertrophy develops. This is manifested by reduced RV ejection fraction, pathological fibrosis, elevated end-diastolic pressure, and increased levels of brain natriuretic peptide (BNP) [2]. Early clinical manifestations are dyspnea, usually accompanied by fatigue, angina, dizziness, edema, and syncope. If left untreated, circulatory dysfunction is exacerbated, leading to organ ischemia, hypoxia, and a series of other complications. In severe cases, RV hypertrophy can lead to cardiac arrhythmias or even sudden death.

Pathological fibrosis of the RV is closely related to RV function, as evidenced by the accumulation of extracellular matrix (ECM) and pathological changes in the collagen network [5]. RV fibrosis is a hallmark of virtually all cardiac diseases, and is particularly common in idiopathic pulmonary hypertension and chronic thromboembolic pulmonary hypertension (CTEPH)-induced RV pressure overload [6]. This reactive fibrosis initially acts as a protective mechanism against RV dilatation. However, in the long term it leads to ventricular stiffness, diastolic dysfunction, and right heart failure (RHF), ultimately becoming the most common cause of death in patients with PH. Cardiac fibroblasts (CF), the primary collagen-producing cells, are activated by mechanical stress, neurohormonal stimuli, and inflammatory mediators. Excessive ECM deposition alters myocardial mechanical properties and increases ventricular stiffness, thereby contributing to RV dysfunction [5]. Although the degree of RV fibrosis correlates with disease severity and prognosis, its therapeutic significance is still under debate. Some studies have shown that RV fibrosis is reversible after mechanical unloading and is ameliorated by pharmacological inhibition, but others have shown that inhibition of pro-fibrotic factors does not improve RV function. In this article, we review the molecular mechanisms underlying the development of RV fibrosis and its clinical relevance in PH, as well as preclinical and clinical intervention studies of RV fibrosis in PH.

## 2. Literature Review

### 2.1 Normal Right Ventricle

RV function depends on the interaction between cardiomyocytes (CM) and mesenchymal stromal cells. The CM is the major functional cell, while mesenchymal stromal cells provide structural support and are involved in synthesis and regulation of the ECM. CF is the major collagen-producing cell, secreting type I and type III collagen to make up the ECM. Collagen synthesis and degradation are balanced by the actions of matrix metalloproteinases (MMPs) and tissue inhibitors of metalloproteinases (TIMPs) [6]. The ECM supports myocardial function by maintaining myocardial segment length, supporting CM alignment and ventricular morphology, transmitting mechanical forces, and promoting diastolic myocardial re-extension [6].

### 2.2 ECM Changes and Fibrosis in Pathological Conditions

Myocardial fibrosis is a common ECM remodeling process in various cardiac diseases. In patients with PH, a certain degree of pressure overload increases collagen formation, eventually leading to excessive collagen deposition. This is accompanied by a high rate of collagen renewal driven by the activation of MMPs and TIMPs, which leads to further imbalance in ECM homeostasis and exacerbates fibrosis [7]. RV diastolic stiffness in PAH was shown to coincide with increased RV contractility (Ees) and force-generating capacity of RV CM (active force). This may represent a compensatory response to increased afterload, but excessive contraction can also impair diastolic function. The elevated diastolic pressure stiffness in the RV results primarily from reduced titin protein phosphorylation, causing an approximately 3-fold increase in myofibrillar rigidity [8]. In patients with idiopathic PAH and CTEPH, RV afterload may increase by up to 5-fold, suggesting that RV pressure overload serves as a common triggering factor [5].

#### 2.2.1 Changes in Collagen Type

PH induces significant shifts in collagen composition. Studies have shown that chronic pressure overload and hypoxia in PH lead to increased synthesis and deposition of type I collagen in both the pulmonary vasculature and RV myocardium, making it a dominant marker of fibrotic remodeling [9]. In contrast, type III collagen exhibits variable changes depending on the disease severity, with some reports indicating it shows a relative reduction in advanced PH, contributing to decreased myocardial elasticity [6].

While type I and type III collagen are the most studied in PH, emerging evidence suggests the potential involvement of other collagen subtypes. Type IV collagen for instance, which is typically associated with basement membranes, may also participate in vascular remodeling during PH progression. Additionally, fibrotic stimuli such as transforming growth factor-β (TGF-β), which is upregulated in PH, can modulate collagen cross-linking and alter the type I/III ratio, further impairing ventricular compliance [6].

#### 2.2.2 Analysis of the Myocardial Remodeling Mechanism

Collagen fiber remodeling and loss of tissue anisotropy are key factors in the transition from adaptive to maladaptive remodeling [3]. Beyond collagen content, the microarchitectural reorganization of collagen fibers—particularly their crimping/slack state and reorientation—plays a pivotal role in determining myocardial mechanical behavior in both PH and heart failure with preserved ejection fraction (HFpEF) [3, 10]. RV adaptation in PH involves myofiber and collagen fiber realignment to mitigate dilation. However, this can transition to maladaptive remodeling when the stiffness exceeds a critical threshold [3].

The pathological transformation in PH stems from a multilevel cascade involving firstly abnormal collagen deposition and increased fiber tautness which alter myocardial matrix properties. This is followed by myofiber realignment that modifies tissue anisotropy, ultimately leading to geometric remodeling [3]. The similar microstructural adaptation patterns observed in both PH-RV and HFpEF-left ventricular (LV) remodeling underscore the universal importance of evaluating collagen architecture, beyond simply measuring its content.

### 2.3 Triggers for RV Fibrosis in PH

**Fig. 1.**
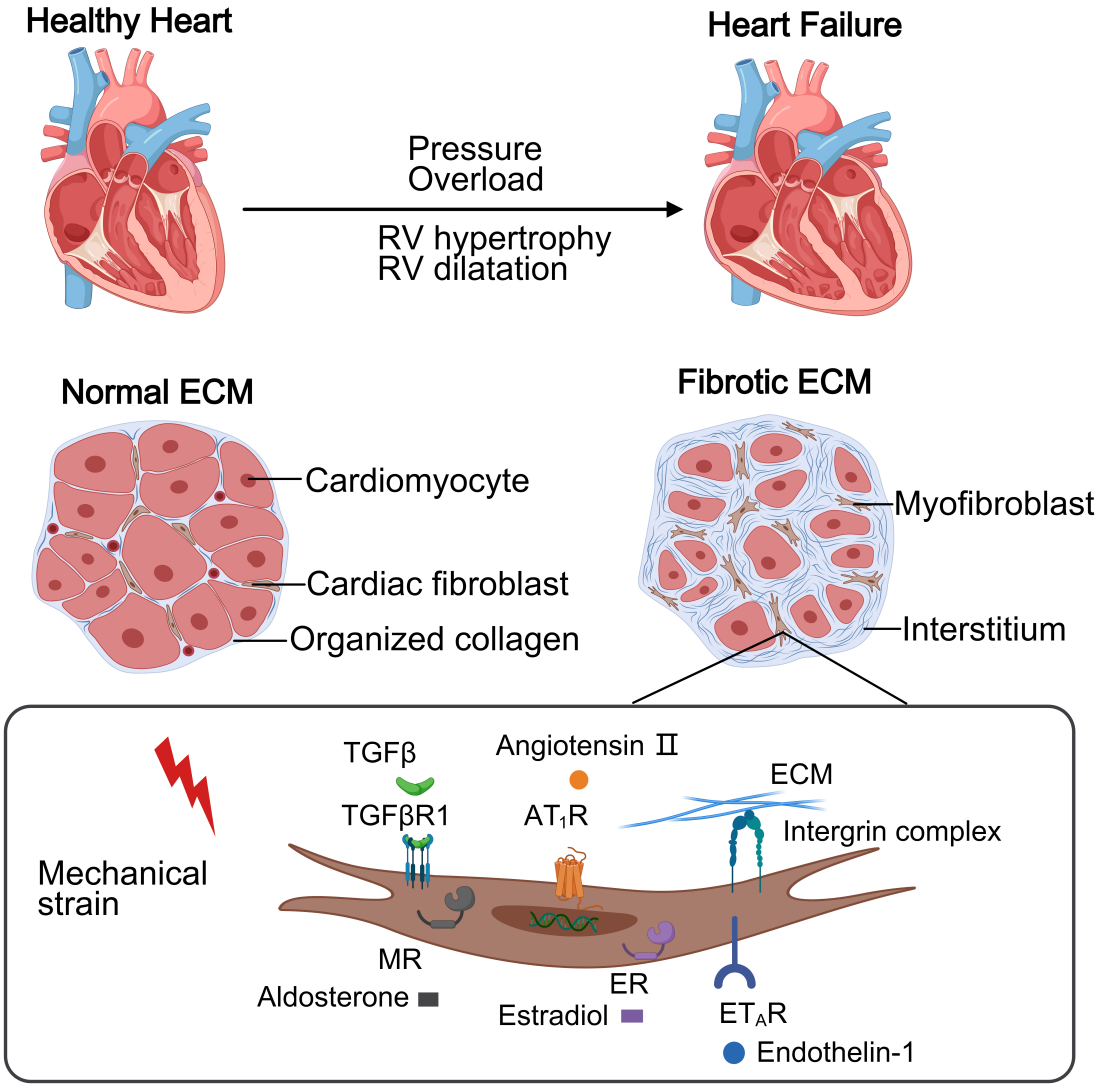
**This schematic contrasts a healthy heart and PH-induced right heart failure**. The normal right ventricular (RV) maintains thin walls with organized collagen extracellular matrix (ECM) supporting cellular homeostasis. In PH, pressure overload causes RV hypertrophy and diffuse fibrosis via excessive collagen deposition. Mechanical stress activates cardiac fibroblasts through receptors, driving collagen production and progressive dysfunction. TGFβR1, transforming growth factor beta receptor 1; AT_1_R, angiotensin II receptor type 1; MR, mineralocorticoid receptor; ER, estrogen receptor; ET_A_R, endothelin-1 receptor type A. Created with MedPeer (www.medpeer.cn).

RV fibrosis in PH patients is influenced by multiple factors, including mechanical stress, neurohormonal systems, ischemia, and inflammation. These factors are interrelated and may act simultaneously [5]. Prolonged stress overload leads to increased fibroblast proliferation and collagen production, dysregulation of integrin expression, release of TGF-β to activate myofibroblasts, upregulation of α-smooth muscle actin (α-SMA), and increased collagen production. CM also respond to mechanical stress by producing TGF-β and angiotensin-II (Ang-II). Increased levels of Galactose lectin-3 (Gal-3) promote TGF-β1-induced cardiac fibrosis by interacting with nicotinamide adenine dinucleotide phosphate oxidase 4 (NOX-4) and NOX-4-derived oxidative stress. Endothelin-1 (ET-1) can mediate RV fibrosis and dysfunction, stimulate fibroblast proliferation, and promote ECM protein synthesis [7]. The endothelial-to-mesenchymal transition (EndMT) is associated with myocardial fibrosis and diastolic dysfunction [7]. Aging is also associated with increased collagen production and decreased collagen degradation [11]. Moreover, gender is a critical variable in ventricular remodeling, with women showing a delayed RV functional decline in PH despite a similar fibrosis burden, possibly due to estrogen-mediated attenuation of collagen cross-linking [12, 13] (Fig. 1).

Research has shown that diffuse RV fibrosis is prevalent among patients with PAH and PH-HFpEF. The RV extracellular volume fraction (ECV) in PAH is closely associated with total pulmonary resistance (TPR), whereas RV fibrosis in PH-HFpEF shows no significant correlation with afterload. Intrinsic myocardial abnormalities such as LV, left atrial (LA) and RV chamber enlargement, increased RV myocardial stiffness, and reduced strain may play a dominant role in the RV ECV of PH-HFpEF. Although TPR was lower in the PH-HFpEF group than in the PAH group, both exhibited similar degrees of RV fibrosis [14].

### 2.4 Clinical Relevance

#### 2.4.1 Assessment and Detection of RV Fibrosis

2.4.1.1 Histological Examination

Endocardial myocardial biopsies, surgically excised cardiac tissue, and autopsy specimens can be used to assess RV fibrosis. However, these methods are all invasive and it is therefore difficult to obtain samples in the early stages of disease. Most studies involve patients with end-stage PAH, and small sample sizes may not fully reflect fibrosis in PH patients with different etiologies and at different stages of disease [5]. Histological examination can assess interstitial and perivascular fibrosis, but may be unable to distinguish between different collagen subtypes, the degree of collagen cross-linking, and changes in the structural integrity of the ECM.

2.4.1.2 Imaging Techniques

Cardiac Magnetic Resonance (CMR) imaging is currently the gold standard and the main non-invasive method to assess RV fibrosis. CMR provides superior spatial resolution for accurate three-dimensional (3D) analysis of myocardial deformation. It is particularly valuable for detecting subtle regional functional abnormalities in patients with PH [1]. CMR-derived strain parameters in CTEPH show significant correlation with ECM remodeling, offering novel mechanistic insights into RV maladaptation [15]. The degree of RV fibrosis measured by CMR correlates with pulmonary hemodynamics, RV function and volume, and adverse clinical outcomes [16]. However, cardiac fibrosis cannot be detected in the early stages of heart failure (HF). Late gadolinium enhancement (LGE) magnetic resonance imaging (MRI) can detect focal fibrosis in the region of the ventricular insertion site, but the dynamic course of fibrosis is more difficult to ascertain. Longitudinal relaxation time (T1) mapping and ECV measurements provide a more comprehensive picture of diffuse fibrosis, which is closely related to RV dysfunction [5].

Diffusion tensor imaging allows the assessment of tissue composition and structure, while enhanced computed tomography (CT) scans, echocardiography, and circulating markers of collagen metabolism have also been used to assess fibrosis [17]. Speckle-tracking echocardiography (STE) has recently proven to be an effective method for assessing RV function. Reduced right ventricular free wall longitudinal strain (RVFWLS) is a predictor of poor prognosis in patients with PH, and has also been shown to correlate with the degree of RV myocardial fibrosis. Compared to pathological results, 3D-RVFWLS is a non-invasive method for the identification of severe myocardial fibrosis in patients with indicators of end-stage HF [18].

Novel molecular imaging techniques, such as enhanced MRI with collagen-targeted contrast agents, or positron emission tomography (PET) imaging with collagen type I -specific probes, are expected to overcome the limitations of existing techniques [5]. To image the heart and lungs, a bimolecular PET-MRI imaging protocol has been developed using a type I collagen-targeted PET probe (^68^Ga-CBP8) and a lysine-targeted fibrogenesis MRI probe (Gd-1,4). This approach can assess cardiopulmonary fibrosis, allow staging and early diagnosis of the disease, as well as monitor the response to treatment. However, its feasibility and clinical value require further research [19]. Fibroblast activation protein inhibitor-42 (FAPI-42) can be detected by PET/CT imaging. A recent PET/CT imaging study reported a higher uptake of FAPI-42 in the RV of PH patients, as well as a progressive increase with the duration of pressure overload. PET/CT with [^18^F]-FAPI-42 can thus be used as a noninvasive tool to accurately assess RV fibrosis and the development of RHF [20].

2.4.1.3 Biomarkers

Collagen triple helix repeat-containing protein 1 (CTHRC1) was reported to be a promising biomarker associated with RV functional impairment and fibrotic remodeling in PH, with particular relevance for monitoring therapeutic response to balloon pulmonary angioplasty in CTEPH [21]. Among the validated markers of ECM turnover, MMP-9 and TIMP-1 levels show robust correlations with disease severity in PAH, reflecting ongoing collagen dysregulation [11]. Advanced imaging biomarkers including ECV quantification and T1 mapping provide early detection of fibrotic changes, with elevated ECV and prolonged T1 relaxation times frequently preceding measurable contractile dysfunction, as evidenced by their dissociation from RV ejection fraction [5, 14]. This temporal pattern suggests the above parameters may serve as sentinel markers of subclinical RV pathology.

Several novel circulating proteins show diagnostic and prognostic potential across the PH spectrum. An increased level of COL18A1/endostatin (ES) was observed early in RV disease progression and showed strong associations with histologically confirmed fibrosis [22]. Furthermore, cartilage intermediate layer protein 1 (CILP-1) appears to regulate myocardial fibrotic responses and may predict incident RV dysfunction in both PH and HF populations [23]. The pleiotropic effects of fibroblast growth factor 23 (FGF-23) extend to maladaptive RV remodeling processes [24]. They are paralleled by systemic indicators such as soluble ST2 and GDF-15 that show particular utility in stratifying the risk of impending RV failure [25].

2.4.1.4 Clinically Relevant Animal Models

Currently, the most commonly used animal models for PH research include the monocrotaline (MCT)-induced model, the Sugen hypoxia (SuHx)-induced model, and the pulmonary artery banding (PAB) model. The experimental animals used include rats, mice, pigs, and sheep. While the PAB model offers valuable insights into RV targeted therapies, it does not reflect changes in pulmonary vascular resistance (PVR) [6]. A dynamic PH with RHF model was developed in sheep. This was achieved by ligating the left pulmonary artery, progressively tightening the main pulmonary artery fascicle and implanting an RV pressure catheter, adjusting the rate of fascicle tightening to control the disease severity and RV phenotype, and assessing the effects of exercise in conjunction with exercise testing. The model successfully induced elevated RV pressures and ventricular remodeling and dysfunction. Moreover, it could induce varying degrees of RHF and fibrosis depending on the rate of fascicle tightening [26].

#### 2.4.2 Relationship of RV Fibrosis to Prognosis

RV fibrosis is strongly associated with poor prognosis in patients with PAH. Decreased longitudinal strain serves as a significant predictor of poor prognosis in PH, with the pathophysiological basis primarily involving three key mechanisms: (1) Myofiber disarray—realignment of collagen and myofibers disrupts normal force transduction, substantially impairing contractile efficiency; (2) Microvascular dysfunction—hypoxia-induced capillary rarefaction exacerbates energy deficiency in longitudinally-oriented subendocardial fibers; and (3) Ventricular-arterial uncoupling—strain abnormalities directly reflect increased RV afterload secondary to elevated PVR, thus accelerating cardiac decompensation [27]. The combined assessment of strain parameters with CMR analysis may have superior predictive value for clinical outcomes compared to conventional RV functional indices [15].

Fibrosis at the ventricular insertion, mainly characterized by increased LGE, T1, and ECV, is an important indicator of poor prognosis. Increased T1 relaxation time and ECV may serve as early markers of PH disease progression. They suggest an early onset of septal shift, and are thus becoming important tools in the risk assessment of PH patients [5]. However, most existing studies are based on patients with end-stage PAH, and hence the prognostic value of these markers in the early stages of disease requires further investigation.

#### 2.4.3 Reversibility of RV Fibrosis

RV fibrosis is a dynamic process, with partial reversibility observed in preclinical models. Reversal of RV hypertrophy, or “reverse remodeling” by targeted therapy, may improve the prognosis of PH. In severe PH, reverse RV remodeling is associated with reduced PVR [28]. RV function has been shown to improve after pulmonary endarterectomy, although RV fibrosis persists in some areas [29]. Mechanical unloading may also lead to partial reversal of RV fibrosis. Treatment with Iloprost can partially reverse established RV fibrosis by inducing collagen degradation and reducing neo-collagen synthesis [5]. A recent study employed 3D deep tissue imaging to compare the RV microvascular network between PAB mice and PH patients [30]. This work revealed complex microvascular remodeling in banded mice, with vessels stably wrapped around hypertrophied CM surfaces. Of note, these changes proved reversible upon the release of banding. In contrast, the microvascular-CM contact in fibrotic regions of the ECM remained impaired. Further investigation of the reversibility of RV fibrosis is therefore needed to optimize treatment strategies.

### 2.5 Treatment Strategy

In recent years, an increasing number of studies have focused on therapeutic strategies that target RV fibrosis to improve RV function and prognosis. However, only a few clinical trials have investigated RV fibrosis (Table 1). Sotatercept, which targets the Bone Morphogenetic Protein Receptor Type 2 (BMPR2)/TGF-β pathway, was found to reduce Right Ventricular End-Diastolic Volume (RVEDV) and RV mass, thus showing promise for reversing RV remodeling and improving fibrosis [31] (Table 2, Ref. [32, 33, 34, 35, 36]). Most drugs reduce pressure overload by targeting the pulmonary vasculature, and preclinical trials have shown that reducing RV fibrosis directly improves RV function. Preclinical therapies that target RV fibrosis are discussed below in the context of the molecular mechanisms that underlie the development of RV fibrosis.

**Table 1.** **Clinical trials targeting RV fibrosis**.

Intervention	Target mechanism	NCT number	Results	Status	Phase
Eplerenone	RAAS	NCT00703352	N/A	Completed	Phase 4
Spironolactone	RAAS	NCT03593317	N/A	Not yet recruiting	Phase 2
		NCT03344159	N/A	Recruiting	Phase 4
Sacubitril/Valsartan	ARNI	NCT04197050	N/A	Not yet recruiting	Phase 4
Trimetazidine	FAO	NCT03273387	No significant reduction in RV fibrosis	Completed	Phase 2/3
Sotatercept	BMPR2/TGF-β	NCT06658522	No significant reduction in RV fibrosis	Not yet recruiting	Phase 4

RAAS, renin-angiotensin-aldosterone system; ARNI, angiotensin receptor-neprilysin inhibitor; FAO, fatty acid oxidation; BMPR, bone morphogenetic protein receptor; N/A, not applicable; NCT, national clinical trial.

**Table 2.** **Preclinical trials targeting RV fibrosis (vasodilatory agents)**.

Target	Therapeutic drug	Animal model	Main result			Ref
			RV Function	RV Fibrosis	PVR	
Prostacyclin analogs	Iloprost	SuHx rat, PAB rat	↑	↓		[32]
sGC stimulation	Riociguat	SuHx rat, PAB rat	↑	↓		[33]
PDE-5 inhibition	Sildenafil	SuHx rat, PAB rat	↑	↓		[34, 35]
AMPK activator	Metformin	MCT rat	↑	↓		[36]

sGC, soluble guanylate cyclase; PDE-5, phosphodiesterase type 5; AMPK, adenosine 5^′^-monophosphate (AMP)-activated protein kinase; MCT, monocrotaline; SuHx, sugen hypoxia; PAB, pulmonary artery banding; ↑, increased; ↓, decreased; PVR, pulmonary vascular resistance.

#### 2.5.1 Renin-Angiotensin-Aldosterone System (RAAS)

The RAAS system plays an important role in the pathogenesis of PAH. Dysregulation of RAAS affects the pulmonary vasculature, and this system is also directly involved in the development of cardiac fibrosis. Pressure overload induces ACE production to generate Ang II. The pro-fibrotic effects of Ang II are associated with activation of TGF-β signaling, while the binding of Ang II to the angiotensin type 1 receptor (AT1R) can also induce pro-fibrotic signaling independently of TGF-β. Activation of AT1R leads to phosphorylation of Smad2 and Smad3 via the extracellular signal-regulated kinase (ERK)/p38 mitogen-activated protein kinase (p38)/c-jun N-terminal ninase (JNK) pathway, which promotes CF activation and collagen synthesis [6]. Blocking of the Ang Ⅱ receptor reduces cardiac fibrosis by attenuating EndMT [7]. Angiotensin signaling releases aldosterone, which induces activation of the salt-receptor and acts as a transcription factor to promote expression of pro-fibrotic genes. Clinical studies of the effects of RAAS inhibitors (Table 3, Ref. [37, 38, 39, 40, 41, 42, 43, 44, 45, 46, 47]) on RV fibrosis have generated great interest [6].

**Table 3.** **Preclinical trials targeting RV fibrosis (inhibition of RAAS and modulation of adrenergic signaling)**.

Target	Therapeutic drug	Animal model	Main result			Ref
			RV Function	RV Fibrosis	PVR	
ARNi	Sacubitril/Valsartan	SuHx rat, PAB mice	↑	↓	↓	[37, 38]
Ang II	ACE2	PAB mice	↑	=		[39]
MR/RAAS	Spironolactone	MCT rat		=	↓	[40]
		Hx mice		↓	↓	[40]
α1A-adrenoceptor, agonism	A61603	Bleomycin mice	↑	↓	=	[41]
		PAB mice	↑	=		[42]
β-blockers	Bisoprolol, Carvedilol, Metoprolol	MCT rat	↑	↓	=	[43, 44, 45]
Reduction of heart rate	Ivabradine	SuHx, MCT, PAB rat	↑	↓	=	[46]
β3-adrenergic receptor agonists	CL316243	Hx mice, SuHx mice	↑	↓	↓	[47]

Ang, angiotensin; ACE, angiotensin-converting enzyme; ↑, increased; ↓, decreased; =, no effect.

#### 2.5.2 Adrenergic Signaling

Abnormalities in the adrenergic signaling pathway have also been reported in patients with PAH. *In vitro* experiments have shown that activation of the β-adrenergic receptor promotes fibroblastogenesis and induces fibrotic remodeling through the phosphoinositide 3-Kinase (PI3K), p38 and ERK pathways via the calcineurin-Nuclear Factor of Activated T-cell (NFAT) pathway. Prolonged β-adrenergic stimulation may lead to RV fibrosis [6]. The β3-adrenoceptor agonist CL316243 reduces RV systolic blood pressure to a similar extent as leucovorin and sildenafil. This agonist has been shown to reverse pulmonary vascular remodeling, reduce RV afterload, and decrease RV hypertrophy and fibrosis in hypoxic models [47] (Table 3, Ref. [37, 38, 39, 40, 41, 42, 43, 44, 45, 46, 47]).

#### 2.5.3 Growth Factor Therapy

Growth factors play a key role in the development of RV fibrosis, with the TGF-β superfamily being the most widely studied pro-fibrotic factor. Increased levels of TGF-β are observed in pressure overloaded RV. The induction of BMP signaling may also have antifibrotic effects [6]. A recent study reported a novel, non-metallic nano-enzyme MMP that attenuates pulmonary vascular remodeling and RV fibrosis in MCT rats by inhibiting the TGF-β1 reactive oxygen species (ROS) signaling pathway and reducing the expression of TGF-β1 and its downstream signaling molecules Smad/3 [48]. It has an excellent *in vivo* safety profile, thus providing a new strategy for the treatment of RV fibrosis. Expression of the transcription factor Forkhead box O3 A (FOXO3A) in myocardial wall tissue gradually decreases with disease progression, whereas the expression of BNP and collagen types I and III increase. It has been suggested that reduced FOXO3A expression may be associated with RV dysfunction (Table 4, Ref. [49, 50, 51, 52, 53, 54, 55, 56, 57, 58]), and is therefore a potential target for intervention in RV myocardial fibrosis [18].

**Table 4.** **Preclinical trials targeting RV fibrosis (growth factors and metabolic modulation)**.

Target	Therapeutic drug	Animal model	Main result			Ref
			RV Function	RV Fibrosis	PVR	
Galectin-3 inhibition	N-acetyllactosamine	PAB mice	=	↓		[49]
TGF-β inhibition	Pirfenidone	SuHx rat	↑	↓	↓	[50]
	Pirfenidone	PAB mice	=	↓		[49]
	Nintedanib	SuHx rat	=	↓	=	[51]
Induction of BMP signaling	FK506	PAB mice, BMPR2 mutant mice	↑	↓		[52]
PDGFR inhibitor	Sorafenib, Sunitinib	MCT rat, PAB rat	↑	↓	↓	[53]
FAO inhibition	Trimetazidine, Ranolazine	PAB rat	↑	↓		[54]
	Ursolic acid	MCT rat	↑	↓		[55]
	Acetazolamide	SuHx rat	↑	↓	↓	[56]
PPARγ activation	Pioglitazone, chrysin	MCT rat, SuHx rats	↑	↓	↓	[57, 58]

PDGFR, platelet-derived growth factor receptor; PPAR, peroxisome proliferator-activated receptor; FK506, tacrolimus; ↑, increased; ↓, decreased; =, no effect.

#### 2.5.4 Compensation of ECM Remodeling 

Excessive deposition and abnormal cross-linking of ECM are important features of RV fibrosis. Lysyl oxidase (LOX) and lysyl oxidase homolog (LOXL2) catalyze collagen cross-linking, and their overexpression can exacerbate fibrosis. Inhibition of LOX/LOXL2 activity reduces collagen cross-linking and attenuates RV fibrosis [11]. The TGFβ1-Snail Family Transcriptional Repressor 1 (Snai1)-LOXL2 axis is central to the regulation of RV fibrosis, and targeting Snai1 inhibits fibrosis, thereby improving PH-RVF [59]. The non-antihypertensive metabolite of chlorosartan, EXP3179, reduces LOX overexpression and increases its activity, thereby preventing collagen cross-linking [11]. Activation of the adenosine A2B receptor (A2BAR) promotes CF proliferation and myofibroblast differentiation, thus exacerbating RV remodeling, but has a lesser effect on collagen production. Blocking A2BAR may be a potential strategy for the attenuation of RV remodeling and RHF [60]. Strategies that target ECM remodeling are still at an early stage and require further investigation.

#### 2.5.5 Metabolic Regulation

2.5.5.1 Metabolic Characteristics of RV Fibrosis

Cardiometabolic abnormalities, particularly the Warburg effect of aerobic glycolysis, are a fundamental pathological feature in the development of RV fibrosis among PH patients [7]. Characteristic metabolic shifts include upregulated glycolysis and glucose oxidation, alongside impaired β-oxidation. These alterations lead to lipotoxicity when the fatty acid supply exceeds the mitochondrial oxidative capacity, with excessive mitochondrial fragmentation disrupting the fibroblast proliferation-apoptosis equilibrium and collectively promoting fibrotic remodeling. Systemic metabolic dysfunction has been identified as a modifiable risk factor for RV failure, with aberrant fatty acid oxidation (FAO) representing a key diagnostic hallmark [61].

2.5.5.2 Metabolic-Targeted Therapeutic Strategies

Excessive protein glycosylation exacerbates RV dysfunction in preclinical PAH models via the suppression of FAO [62]. Chrysin (CH) has multi-target effects in SU5416/hypoxia-induced PAH models. It ameliorates cardiac fibrosis, RV hypertrophy and PH through the coordinated regulation of mitochondrial biogenesis, energy metabolism, and gene expression [58]. Metformin is another pleiotropic agent, with phase II trial data (NCT01884051) indicating RV functional improvement and modulation of lipid metabolism in PH patients (Table 4, Ref. [49, 50, 51, 52, 53, 54, 55, 56, 57, 58]). Mechanistic studies in MCT-treated rats demonstrate its capacity to activate adenosine 5^′^-monophosphate (AMP)-activated protein kinase (AMPK) signaling, enhance nitric oxide bioavailability, preserve contractile function, and prevent fibrotic remodeling [36].

#### 2.5.6 Anti-inflammatory and Antioxidant Therapy

2.5.6.1 Pathological Mechanisms

The pathogenesis of RV fibrosis induced by pressure-overload involves synergistic interactions between chronic inflammatory activation and mechano-sensitive ROS generation [6, 7]. Mechanical stretching triggers inflammatory cascades while simultaneously increasing oxidative stress, thereby creating a self-perpetuating cycle that drives fibrotic progression.

2.5.6.2 Therapeutic Intervention

Dihydromyricetin reduces inflammatory responses and ameliorates fibrosis and RV hypertrophy by inhibiting cellular pyroptosis mediated by the chemokine-like factor 1 (CKLF1)/C-C motif chemokine receptor 5 (CCR5) axis [63]. Lingguizhugan decoction [64] and notopterol from Qiang-Huo [65] may improve RV fibrosis and dysfunction by modulating multiple inflammatory pathways and immune cell activities [64]. Tripotassium hydroxycitrate hydrate reduces inflammation and oxidative stress levels and effectively attenuates RV fibrosis and pulmonary vascular remodeling [66]. Melatonin attenuates CM hypertrophy and mitochondrial oxidative stress and improves RV fibrosis in rats by activating the Mst1-Nrf2 signaling pathway [67] (Table 5, Ref. [63, 64, 65, 66, 67, 68, 69, 70, 71, 72, 73, 74, 75, 76, 77, 78, 79, 80, 81, 82, 83, 84]).

**Table 5.** **Preclinical trials targeting RV fibrosis (anti-inflammatory and antioxidant)**.

Target	Therapeutic drug	Animal model	Main result			Ref
			RV Function	RV Fibrosis	PVR	
Anti-inflammatory; AKT/ERK inhibition	Celastrol	MCT rat/Hx mouse/SuHx rat	↑	↓	↓	[68, 69]
Anti-inflammatory, Nrf2	Sulforaphane	SuHx mice	↑	↓	↓	[70]
Nrf2 induction	Protandim	SuHx rat	↑	↓	=	[71]
Anti-inflammatory; ROCK inhibition	Tsantan Sumtang	Hx rat	↑	↓	↓	[72]
Anti-inflammatory, P38/MAPK	Magnesium lithospermate B; PH797804	PAB mice	↑	↓		[73, 74]
Anti-inflammatory: TLR9/ NFκB	E6446/Pyrrolidinedithiocarbamate	PAB rat	↑	↓		[75]
ASK1/p38/JNK inhibition	GS-444217	MCT rats, SuHx rat, PAB mice	↑	↓		[76]
AKT inhibition	Nitrite	PAB mice	↑	↓		[77]
Anti-Inflammatory	Perillyl alcohol/quercetin/berberberine, Dihydromyricetinn, Lingguizhugan decoction, Notopterol	MCT rat	↑	↓		[63, 64, 65, 78]
Anti-Inflammatory	Sevoflurane, 1,8-Cineole; Compound X	MCT rat	↑	↓	↓	[79, 80, 81]
Anti-inflammatory/antioxidant	EUK-134, Fluvoxamine	MCT rat	↑	↓	=	[82, 83]
Anti-Inflammatory	hydroxycitric acid tripotassium hydrate	MCT rat, Hx rat	↑	↓		[66]
Antioxidant, activation of Mst1-Nrf2 pathway	Melatonin	MCT rat	↑	↓		[67]
Anti-Inflammatory	Vagal nerve stimulation	PAB rat	↑	↓		[84]

AKT, protein kinase B; ERK, extracellular regulated protein kinases; Nrf2, nuclear factor erythroid-derived 2-like; ROCK, rho-associated kinase; MAPK, mitogen-activated protein kinase; TLR, toll-like receptor; NFκB, nuclear factor kappa-B; ASK1, apoptosis signal-regulating kinase 1; JNK, c-Jun N-terminal kinase; Mst1, macrophage stimulating 1; ↑, increased; ↓, decreased; =, no effect.

Non-pharmacological interventions also show promise in attenuating disease progression. Diet-induced ketosis improves RV function, inhibits NOD-like receptor protein 3 (NLRP3) inflammatory vesicle activation, and counteracts RV fibrosis [85]. Swimming exercise has also been shown to improve RV structural remodeling and dysfunction, thereby reducing inflammation by improving systemic and RV insulin sensitivity [86].

#### 2.5.7 Other Treatments

The experimental field of non-coding RNAs in the treatment of RV fibrosis is still very limited (Table 6, Ref. [87, 88, 89, 90, 91, 92, 93, 94, 95, 96, 97, 98, 99, 100, 101, 102]). Genes associated with the epithelial-mesenchymal transition and EndMT are significantly enriched in RHF [6], and stem cell administration may be an option for targeting RV fibrosis. Human induced pluripotent stem cell-derived myocardium patch transplantation improves RV function, inhibits ventricular fibrosis, and increases capillary density, thus warranting further clinical studies [99].

**Table 6.** **Preclinical trials targeting RV fibrosis (other treatments)**.

Target	Therapeutic drug	Animal model	Main result			Ref
			RV Function	RV Fibrosis	PVR	
Serotonin signaling antagonists	Terguride, SB204741	PAB mice	↑	↓		[87]
nAChR inhibition	Mecamylamine	SuHx rat	↑	↓	=	[88]
ER	17β-estradiol	MCT rat, SuHx rat	↑	↓	↓	[89, 90]
ROCKs and STAT3 inhibition	Dehydroepiandrosterone	SuHx rat	↑	↓	↓	[91]
SGLT2 inhibition	Empagliflozin, Canagliflozin	MCT rat	↑	↓	↓	[92, 93]
Genetics	H19 Gapmer	MCT rat, PAB rat	↑	↓	=	[94]
Stem cell therapy	Umbilical cord blood mononuclear cells	PAB mice	↑	↓		[95]
	pediatric cardiac progenitor cells	PAB rat	↑	↓		[96]
	Mesenchymal stem cells	PAB pig, SuHx rat	↑	↓		[97, 98]
	Human induced pluripotent stem cells	PAB rat	↑	↓		[99]
CaSR	NPS2143	MCT rat, Hx mouse	↑	↓	↓	[100]
Genetics	siRNA AP-1	MCT rat	↑	↓		[101]
HMOX1/GSH inhibition	Ferrostatin-1	MCT rat	↑	↓		[102]

nAChR, nicotinic acetylcholine receptor; ER, estrogen receptor; STAT, signal transducer and activator of transcription; SGLT2, sodium-glucose cotransporter 2; CaSR, Ca^2+^-sensing receptor; HMOX1, heme oxygenase 1; GSH, glutathione r-glutamyl cysteinyl +glycine; ↑, increased; ↓, decreased; =, no effect.

#### 2.5.8 Indirect Treatments

Several interventions have been shown to indirectly reduce RV fibrosis by improving pulmonary vascular remodeling. A novel lysosomal autophagy inhibitor, ROC-325, was effective in preventing MCT- and Sugen5416/hypoxia-induced PH, vascular remodeling, and RV hypertrophy, fibrosis and dysfunction. The mechanism may be related to inhibition of autophagy, induction of endothelial nitric oxide synthase activity, reduction in the levels of Hypoxia-Inducible Factor 1 Alpha (HIF-1α) and Hypoxia-Inducible Factor 2 Alpha (HIF-2α), and increased NO production [103]. Pharmacological inhibition of Ang II signaling by diminazene reduced both PVR and RV fibrosis, with possible indirect cardioprotective effects [6].

A novel and highly selective inhibitor of platelet-derived growth factor receptor (PDGFR), WQ-C-401, was shown to decrease collagen I synthesis and increase α-SMA expression in pulmonary artery smooth muscle cells, inhibit pulmonary vascular remodeling by reducing muscle formation and fibrosis, and attenuate RV hypertrophy in MCT rats [104]. Salidroside reduced the mean pulmonary arterial pressure and ameliorated RV hypertrophy, collagen deposition, and fibrosis in PAH rats by modulating arginine metabolism, increasing NO synthesis, and improving pulmonary vascular remodeling [105]. Treatments involving vagus nerve stimulation [84] and noninvasive focused ultrasound of the spleen [106] were shown to significantly reduce RV systolic blood pressure and ameliorate RV fibrosis in a rat model of PAH. However, it is unclear whether this is a direct effect on the RV, or a secondary alteration after afterload reduction.

## 3. Conclusions

RV fibrosis due to PH is a complex process in which the clinical significance and therapeutic strategies remain to be thoroughly elucidated. Emerging evidence suggests that RV dysfunction and PH-like hemodynamics may persist in diverse clinical contexts [107]. Some patients with cardiopulmonary injury show characteristic symptoms such as fatigue and exertional palpitations, accompanied by mild PH and significant RV systolic dysfunction. Notably, symptom resolution often parallels RV functional recovery, suggesting a potentially reversible process. These observations highlight the fact that impairment of RV pulmonary circulation is a common pathological feature across multiple disease states.

RV fibrosis appears to have a dual role in PAH. In the early stages, it is an adaptive response of the RV to pressure overload and allows structural integrity to be maintained. However, as the disease progresses, excessive fibrosis leads to RV dysfunction and ultimately to RHF. The transition from an adaptive to a maladaptive response is dynamic, and some fibrosis may be reversible. Current studies have focused on the ventricular insertion site. Reduced longitudinal strain in the RV free wall has been shown to correlate with the degree of RV myocardial fibrosis and may serve as a marker of poor prognosis in patients with PH [18]. Imaging techniques, particularly T1 mapping and ECV measurements, are valuable in assessing RV fibrosis and may be important indicators for early diagnosis and prognosis [5]. Novel molecular imaging techniques and biomarker studies are also being refined to provide new tools for staging and early diagnosis of the disease, as well as for monitoring treatment response.

Chronic mechanical stress-induced CF activation and chronic inflammation are key underlying factors in the mechanism of RV fibrosis. CF senses mechanical stress and initiates complex molecular signaling pathways that lead to ECM generation and remodeling. Chronic inflammation further promotes CF proliferation and activation and exacerbates collagen deposition [6]. Most of the current preclinical trials have failed to fully distinguish the effects of interventions on RV from those on PVR, posing a challenge in the interpretion of results. The timing of antifibrotic therapy is also critical, and a combination of noninvasive imaging and circulating biomarkers is needed to guide treatment and to monitor efficacy. Further studies are needed to better elucidate the molecular mechanisms of RV fibrosis and to develop more effective antifibrotic drugs. The timing of treatment needs to be optimized and new imaging techniques and biomarkers developed in order to better assess RV fibrosis and its relationship with clinical outcomes. Multidisciplinary collaboration will be essential for the advancement of RV fibrosis research.

### Limitations

While this review synthesizes the current evidence on RV fibrosis mechanisms and therapies, several limitations should be acknowledged. First, translational challenges exist between preclinical animal models and human pathophysiology, particularly regarding cross-specific differences in collagen metabolism and drug responses. Second, long-term efficacy and safety data are lacking for many investigational agents. These gaps highlight the need for standardized large-animal models, longer-term randomized controlled trials, and dedicated studies on personalized therapeutic approaches.
